# Stable overexpression of the epithelial sodium channel alpha subunit reduces migration and proliferation in breast cancer cells

**DOI:** 10.1007/s10549-025-07667-w

**Published:** 2025-04-12

**Authors:** Sarah R. A. McQueen, Wey Qi Chin, Heather E. Cunliffe, Fiona J. McDonald

**Affiliations:** 1 Department of Physiology, School of Biomedical Sciences, University of Otago, PO Box 56, Dunedin, 9054 New Zealand; 2 Department of Pathology, Dunedin School of Medicine, University of Otago, Dunedin, New Zealand

**Keywords:** Breast cancer, Epithelial sodium channel (ENaC), Proliferation, Migration

## Abstract

**Purpose:**

Breast cancer is the most common cancer diagnosed in women worldwide. Ion channels have emerged as novel regulators of cancer cell functions, including proliferation and migration. The epithelial sodium channel (ENaC) has a key role in blood pressure regulation, and ENaC levels affect the characteristics of several types of cancer. In breast cancer, a role for αENaC has not been investigated in migration previously nor the effect of stable overexpression of αENaC on proliferation.

**Methods:**

Correlations of the mRNA levels for the four ENaC subunits and breast cancer survival outcomes were assessed in publicly available data and the association between αENaC and migration-related genes. Three isogenic monoclonal derivatives of MDA-MB-231 breast cancer cell lines were created with stable αENaC overexpression. Migration assays (scratch wound assay and Boyden chamber assays) and a proliferation assay (EdU) were used to determine the effect of αENaC overexpression compared to control MDA-MB-231 cells.

**Results:**

Higher α- or δENaC expression was correlated with improved patient survival. Higher αENaC expression correlated with lower expression of migration-associated genes. Stable overexpression of αENaC in MDA-MB-231 cells resulted in reduced in vitro migration and proliferation of all three clones compared to parental control cells.

**Conclusion:**

Higher αENaC expression correlates with improved patient outcomes, and overexpression in breast cancer cells reduces both cell migration and proliferation. These results highlight the possibility of ENaC as a target for future breast cancer treatments.

**Supplementary Information:**

The online version contains supplementary material available at 10.1007/s10549-025-07667-w.

## Background

Breast cancer has a significant burden of disease globally as it is the most diagnosed cancer in women with 2.3 million new cases of breast cancer diagnosed in 2020 [1]. Over 600,000 deaths are globally attributed to breast cancer annually [2]. Metastasis to visceral organs and the brain is implicated in the vast majority of breast cancer-related deaths [3]. To achieve metastasis, the cells undergo processes, such as epithelial–mesenchymal transition (EMT), migration, and proliferation. An area of research that has expanded dramatically in recent years is the possibility of ion channels having a role in regulating these processes.

Ion channels are proteins that allow for the passage of ions in and out of cells and, in normal cells, they function to control ion concentrations and regulate cell volume; therefore, research has begun to determine if ion channels have a role in cancer cells to regulate the cell processes to achieve metastasis [4]. A number of ion channels with varying substrates, including Na^+^, have been identified to have an impact on cancer cell characteristics [5]. The role that the epithelial sodium channel may have in cancer cells has not yet been fully established, with some evidence pointing to expression having a beneficial outcome in some cancers and being detrimental in others [6, 7].

The epithelial sodium channel (ENaC) is a heterotrimeric protein channel composed of three subunits, predominantly α, β, and γENaC, with a fourth δENaC subunit also present in some mammals that can replace αENaC in the heterotrimer [6]. The ENaC subunits are encoded by their respective genes: *SCNN1A*, *SCNN1B*, *SCNN1G*, and *SCNN1D* [8]. A classical role of ENaC is the regulation of Na^+^ reabsorption in the kidney as Na^+^ ions will flow down their concentration gradient into a cell through ENaC [9], which is critical in the homeostasis of water in the body and subsequently, blood pressure regulation. Other non-classical roles of ENaC have been identified, including functioning as a mechanosensor to respond to cues from a cell’s external environment to influence cell shape and stiffness [10, 11].

ENaC expression has been identified to be changed in several types of cancer and changes in ENaC activity and expression have been reported to affect a wide range of cancer cells’ ability to migrate or proliferate [12–18]. In breast cancer, studies have shown that the expression of the αENaC gene expression changes with p53 mutation, ER status and histological grade [18, 19]. Our previous work showed that transient upregulation of ENaC expression levels in breast cancer cell lines decreased proliferation ability of these cells, and we identified that upregulation of αENaC alone decreased the proliferation of breast cancer cells [18]. From this evidence, we hypothesized that a permanent increase in expression of αENaC in a migratory triple-negative breast cancer (TNBC) cell line would reduce their migration ability.

In this paper, we report that the subunits of ENaC show differential correlations with breast cancer survival. Additionally, we report the development of monoclonal MDA-MB-231 cell lines that express significantly higher levels of αENaC overexpression compared to a control MDA-MB-231 cell line. Overexpression of ENaC in these cell lines significantly reduces migration and proliferation compared to the control breast cancer cells.

## Materials and methods

### Cell culture

MDA-MB-231 and T-47D cells were maintained in RPMI-1640 media with 10% FBS (Thermo Fisher), 2-mM l-glutamine, and 10,000 units/mL penicillin and 10,000 μg/mL streptomycin (Thermo Fisher). MDA-MB-231 cell media were additionally supplemented with 2-g/L glucose and 1.5-g/L sodium bicarbonate. T-47D cell media were supplemented with 1-mM sodium pyruvate, 4.5-g/L glucose, 1.5-g/L sodium bicarbonate, and 10-mM HEPES. Cells were grown in a humidified cell culture incubator at 37 °C with 5% CO_2_. To create breast cancer cell lines with stable overexpression of αENaC, Lipofectamine™-3000 (Thermo Fisher) was used to transfect MDA-MB-231 cells with plasmid DNA containing the αENaC gene (Origene Technologies) as previously described [20]. Control cells were transfected with pcDNA3.1 plasmid. The cells were incubated with 700-μg/mL neomycin (G418 disulfate salt, Sigma-Aldrich) for two weeks to select cells with genomic integration of the αENaC gene. Single-cell expansion was then undertaken to achieve monoclonal populations of cells that were overexpressing αENaC. Transfected cells were maintained in 700 μg/mL of neomycin.

### Kaplan–Meier plots

Survival analysis was performed using the breast cancer mRNA dataset on Kaplan–Meier Plotter available at https://kmplot.com/analysis/index.php?p=service. This dataset contains survival information for 4929 breast cancer patients [21, 22]. The target genes entered were the genes that code for the four ENaC subunits.

### Gene expression analysis and migration metagene

αENaC gene expression analysis in the SCAN-B dataset was undertaken as described previously [18]. All in silico analysis was performed in R version 3.6.3. A migration metagene was constructed from a migration-associated gene set, “Cell_Migration” taken from the Molecular Signatures database [23]. All the genes that were associated with migration were represented by the migration metagene numerically ranging from 0 to 1.

### Western blot

Whole cell protein lysate was extracted using lysis buffer (TBS: 50-mM Tris, 150-mM NaCl (pH 7.4), plus 1% Triton X-100, 1 × Complete protease inhibitor cocktail (Roche)), and concentration was determined by DC Protein assay kit (Bio-Rad). Samples were separated by 10% SDS-PAGE and the protein was transferred to PVDF membranes (Sigma-Aldrich) for 2 h at 45 mA (Hoefer Semiphor, Semi-dry transfer unit). Following blocking in 5% skim milk in wash buffer (TBS + 0.1% Tween-20), the membrane was incubated with the appropriate primary antibody (αENaC (1:1000, cat no: SPC-403, StressMarq) or beta-actin (1:5000, cat no: A5441, Sigma-Aldrich) overnight at 4 °C. After washing, membranes were incubated with secondary antibodies (goat anti-rabbit-HRP (1:10,000, cat no: A6154, Sigma-Aldrich) or goat anti-mouse-HRP (1:10,000, cat no: A5441, Sigma-Aldrich)) for 1 h at room temperature. Protein bands were visualized using ECL (GE Healthcare) on a ChemiDoc™ imager (Bio-Rad) or on CL-XPosure autoradiographic film (Thermo Scientific) and developed. To quantify the protein signal, densitometry was undertaken using ImageJ software and protein concentration was normalized to beta-actin.

### RNA extraction and RTqPCR

Total RNA was extracted from cells using the PureLink® RNA Mini Kit (Cat No 12183018A, Life Technologies) following the manufacturer’s instructions. Total RNA was reverse transcribed using a commercial kit (PrimeScript, Medi’Ray, New Zealand). RNA (1 μg) and gene-specific primers (Sigma) were used for real-time PCR with SYBR Premix Ex Taq (Tli RNase H Plus) ROX plus (Medi’Ray) and a Bio-Rad CFX connect Real-Time system (Bio-Rad). The sequences of primers used are as follows: αENaC, forward, 5’- GGGTACTGCTACTATAAGCTC -3’ and reverse, 5’-TTGACGGTGTAATTGTTCTG-3’; βENaC, forward, 5’-CTGGTCCTTATTGATGAACG-3’ and reverse, 5’-ATAGTCCATGGCCATTTTG-3’; γENaC, forward, 5’-CTTCTATACTGTCTCAGTTTCC-3’, and reverse, 5’-TGTACTTGTAGGGGTTGATG-3’; and GAPDH, forward, 5’-ACAGTTGCATGTAGACC-3’ and reverse, 5’-TTGAGCACAGGGTACTTTA-3’. mRNA expression was normalized to the internal control GAPDH. Data are represented as relative dCT expression.

### Scratch wound assay for migration

Cells (control and αENaC overexpressed clones) were seeded into 6-well plates and incubated until a monolayer was achieved. The cells were scratched with a pipette tip and time point 0 images were taken. The cells were then incubated for 24 h with images taken at 1, 3, 5, 8, 12, and 24-h post-scratch. Images were analyzed using the MRI_Wound_Healing _Tool on ImageJ to determine the percentage of the area remaining uncovered in the scratched area compared to time point 0 h.

### Boyden chamber assay for migration

Cells (control and αENaC overexpressed clones) were pipetted into the upper chamber of the Boyden chamber cup (8 µM diameter membrane pores) in serum-free media, and full media was placed in the lower well. The wells were incubated for 5 h to allow cell migration, after which cells were fixed in methanol and stained with 2% crystal violet. The membranes were imaged on an inverted microscope, 8 images were taken per cup, and quantification was completed using ImageJ. The images were converted to Black and White and a ratio of black:white pixels was generated for quantification.

### EdU for proliferation

Cells (3 × 10^5^) seeded on two 13-mm diameter coverslips per 35 mm^2^ plate and were incubated with EdU for 18 h at 37 °C, and then fixed with 4% paraformaldehyde. Then, cells were permeabilized with 0.5% Triton X-100 in PBS and then incubated with a click-chemistry cocktail as per the manufacturer’s instructions (EdU Click kit, Baseclick). Cells were then counterstained with DAPI and visualized with a Nikon A1 Inverted Confocal (Otago Micro and Nanoscale Imaging Unit, OMNI).

### Statistical analysis

Statistical analyses (predominately one-way or two-way ANOVA with post hoc tests) were conducted using GraphPad Prism unless otherwise stated. ‘N’ indicates the number of independent biological trials and a *p* value of < 0.05 was considered statistically significant, and ***p < 0.001 **p < 0.01, and *p < 0.05.

## Results

### αENaC and δENaC expression correlates with improved breast cancer patient survival and high αENaC expression predicts lower expression of migration-associated genes

**Fig. 1:**
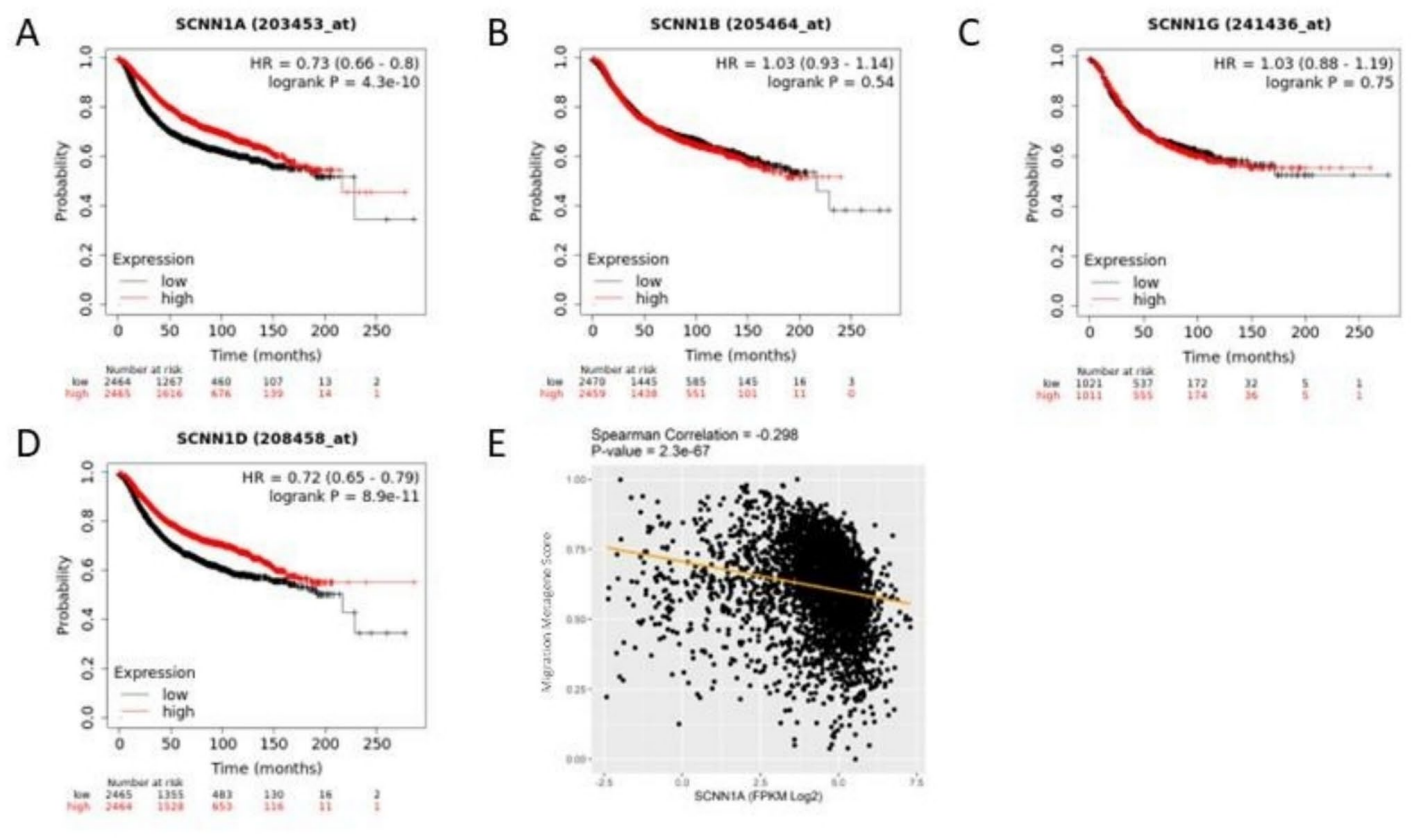
Correlations between ENaC subunits and survival or migration. **A**-**D** Survival analysis with the genes of the four subunits of ENaC ((**A**) *SCNN1A:* alpha, (**B**) *SCNN1B*: beta, (**C**) *SCNN1G*: gamma, and (**D**) *SCNN1D*: delta). Red denotes high expression of the gene of interest, and black is the low expression. Kaplan–Meier plots created using KM plotter for breast cancer [21, 22]. **E** Scatter plot showing the correlation between αENaC mRNA level and expression of migration metagene in SCAN-B dataset. Spearman rank method was used to calculate correlation, with the orange line showing the line of best fit

As there are four ENaC subunits [8], we wanted to confirm if the expression levels of all subunits had a correlation with patient survival. We found that the mRNA expression level of the αENaC and δENaC subunits correlate with differential prognosis in breast cancer patients, however this was not seen with the βENaC or γENaC subunits. Possible correlations in mRNA levels of αENaC, βENaC, γENaC, and δENaC with overall breast cancer patient survival was examined using KM plotter [21, 22], that allows analysis of mRNA expression from ~ 4000 breast cancer patients. Figure 1 A shows that patients with high αENaC mRNA levels have significantly improved survival time compared to patients with low expression of αENaC. Similarly, improved survival time was also seen in those with high δENaC expression (Fig. 1D). By comparison, there was no correlation of mRNA levels for β- (Fig. 1B) or γENaC (Fig. 1C) with patient survival time.

We had previously shown that a metagene of proliferation genes correlated with *SCNN1A* expression [18] and a similar method was used here to compare *SCCN1A *with migration-associated genes, as migration is another important component needed for cells to achieve metastasis. The correlation between *SCNN1A* expression in breast cancer patients and migration gene profile was examined in the SCAN-B data-set. This analysis showed that as expression of αENaC increased the migration metagene score decreased demonstrating that higher αENaC mRNA expression significantly correlates with a lower migration profile (Fig. 1E). This suggests a role for αENaC in breast cancer cell migration; however, we note that the correlation is weak.

### Differential αENaC protein expression in breast cancer cell lines

The alpha subunit of ENaC is found in many tissues around the body, including in mammary tissues [24], and differential mRNA expression has been shown in cancerous breast cell lines [18] but not confirmed at a protein level. Consistent with our previous results that showed a reduction of αENaC mRNA expression in more migratory breast cancer cell lines [18], we report here that αENaC protein level is significantly reduced in the migratory MDA-MB-231 breast cancer cell line compared to the T-47D breast cancer cell line (Fig. 1A, B, unpaired *t* test, *p* < 0.001, *n* = 5).

**Fig. 2:**
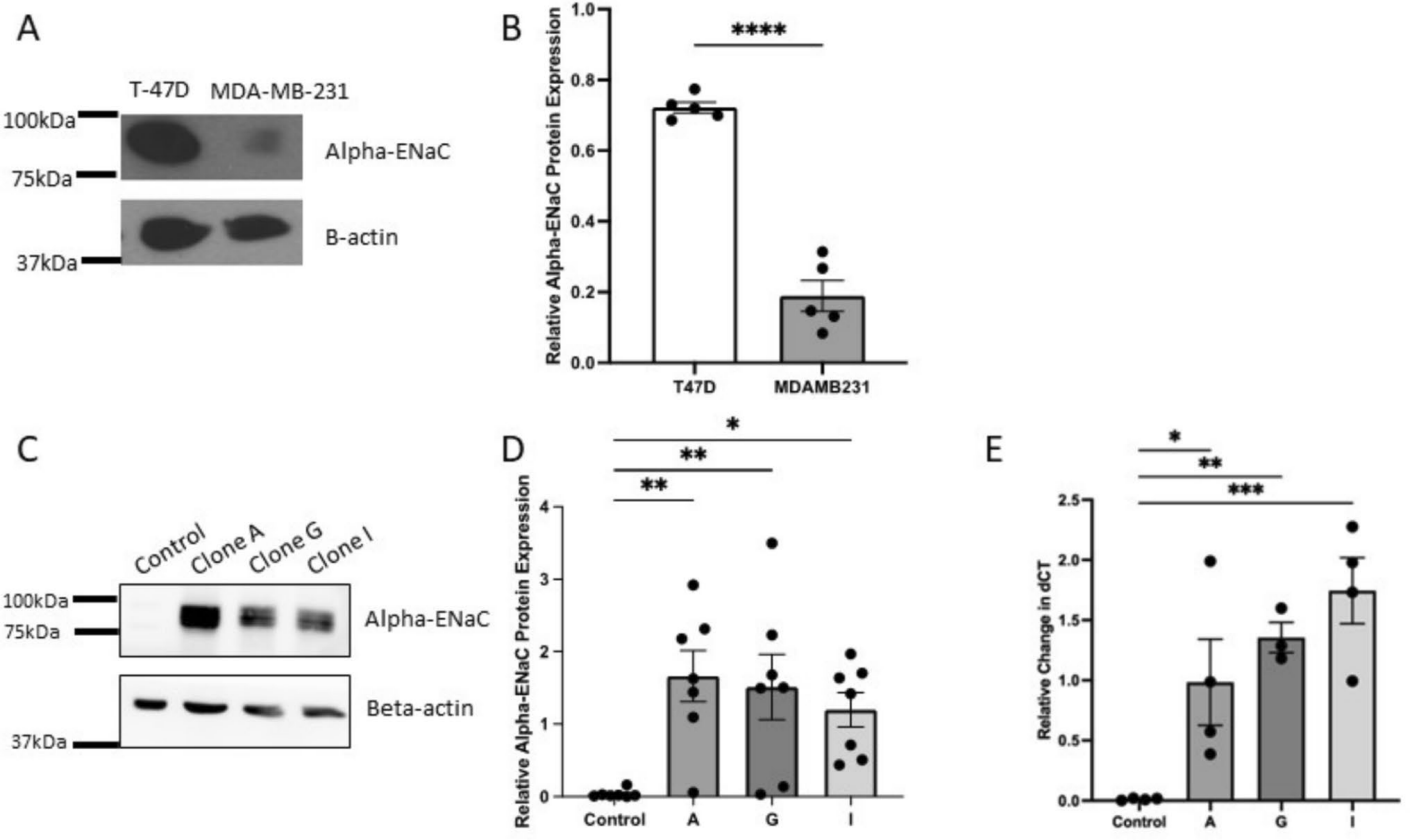
αENaC protein expression in breast cancer cell lines and stable αENaC-overexpressing clones. **A** Representative western blot showing protein expression of αENaC and beta-actin as a loading control for T-47D and MDA-MB-231 cell lines. **B** Semi-quantitative densitometry using ImageJ was undertaken to compare the protein expression of αENaC relative to the loading control of beta-actin. Student’s *t* test performed ****p < 0.001, *N* = 5. **C** Representative Western blot showed an increase in αENaC protein expression in three MDA-MB-231 clones, visualized with anti-αENaC antibody and with beta-actin used as a loading control. Lane 1: control, 2: clone A, 3: clone G, and 4: clone I. **D** αENaC protein bands were semi-quantified using densitometry using ImageJ, and αENaC bands were normalized to beta-actin. One-way ANOVA was performed with Dunnett’s multiple comparisons test, **p < 0.01, *p < 0.05. Data shown as mean ± SEM. *N* = 7. **E** αENaC mRNA expression levels measured by quantitative RT-PCR in control and αENaC-overexpressing clones. One-way ANOVA performed with Dunnett’s multiple comparisons test, ***p < 0.001 **p < 0.01, *p < 0.05, *N* = 4

Since the MDA-MB-231 cell line has low αENaC expression, these cells were used to develop stable cell lines that overexpress αENaC to assess the effect of stable overexpression of αENaC on cell migration and proliferation. Transfection of a plasmid containing αENaC and selection via neomycin was undertaken, followed by cloning to obtain monoclonal populations. Of these clones, three labeled A, G, and I showed consistently increased αENaC protein expression compared to cells transfected with the control plasmid (Fig. 2C). All three of the αENaC overexpressing clones were confirmed to have a significant increase in αENaC protein compared to control cells (Fig. 2D) (One-way ANOVA with post hoc test, *p < 0.05, **p < 0.01, *N* = 7). The mRNA expression of αENaC was also confirmed to be significantly increased in all three clones when compared to the control (Fig. 2E). The mRNA expression of βENaC and γENaC subunits in the clones with αENaC mRNA overexpression did not significantly change (data not shown).

Up or down-regulation of some ion channels has been linked to changes in the epithelial-mesenchymal transition pathway through changes in expression of specific EMT markers [4]. To test if stable overexpression of αENaC altered the levels of three typical EMT markers we quantified the mRNA levels of E-cadherin, N-cadherin, and vimentin mRNA in the control and αENaC overexpressing cell lines using qRT-PCR. Stable overexpression of αENaC did not significantly alter the mRNA levels of E-cadherin, N-cadherin, or vimentin, see supplementary data. At the protein level stable overexpression of αENaC did not result in E-cadherin detection in the cells (wildtype MDA-MB-231 cells do not express E-cadherin). Similarly, the protein levels of N-cadherin and vimentin were not significantly changed, see supplementary data. The results suggest that these EMT markers are not involved in α-ENaC’s role in inhibiting cell proliferation and migration.

### αENaC overexpression in MDA-MB-231 cells results in reduced migration

**Fig. 3:**
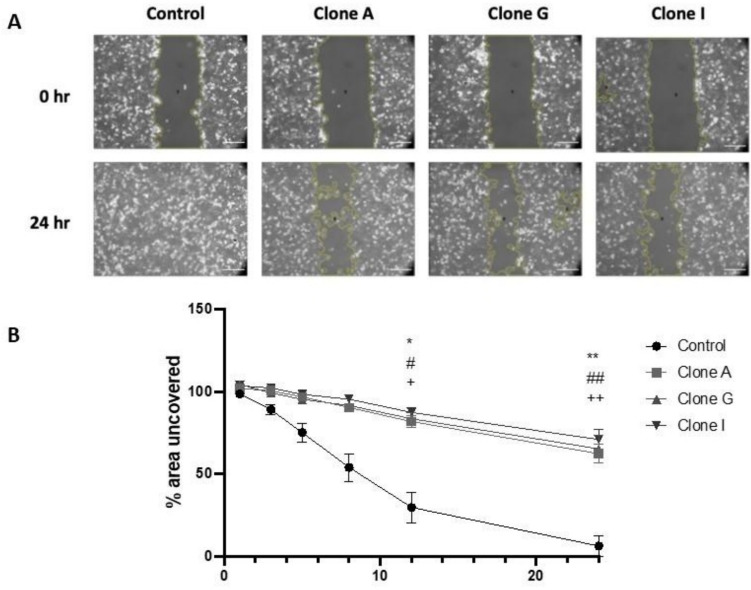
Stable overexpression of αENaC reduces the migration ability of MDA-MB-231 cells. **A** Representative images of control, A, G, and I clone MDA-MB-231 breast cancer cells at time points 0 h and 24 h. Scale bar 200 μm. The yellow outlines indicate the uncovered area used for quantification using ImageJ. **B** All clones showed a statistically significant difference at time points 12 and 24 h in the percentage of area of the scratch that remained uncovered, compared to the control. Data are shown as mean ± SEM. Two-way ANOVA with Tukey’s post hoc test, resulted in a statistically significant difference, * for clone A, # clone G, + clone I, * # + *p* < 0.05, ** ## +  + *p* < 0.01, *N* = 4

Cell migration is important in the process in order for cell metastasis to occur, and some evidence in cancer types other than breast cancer has shown changing ENaC expression has an impact on the cancer cells’ ability to migrate [12, 17, 25, 26]. Using a scratch assay to examine migration, compared to the control MDA-MB-231 cells, all three αENaC overexpression clones had reduced migration ability, evidenced by none of the clones covering the scratched area in 24 h, whereas the control cells did cover the area. (Fig. 3A). A significant reduction of migration ability was observed at both 12- and 24-h post-scratch when comparing all of the αENaC overexpressing clones to control (two-way ANOVA with post hoc test, *p < 0.05, **p < 0.01) (Fig. 3B).

**Fig. 4:**
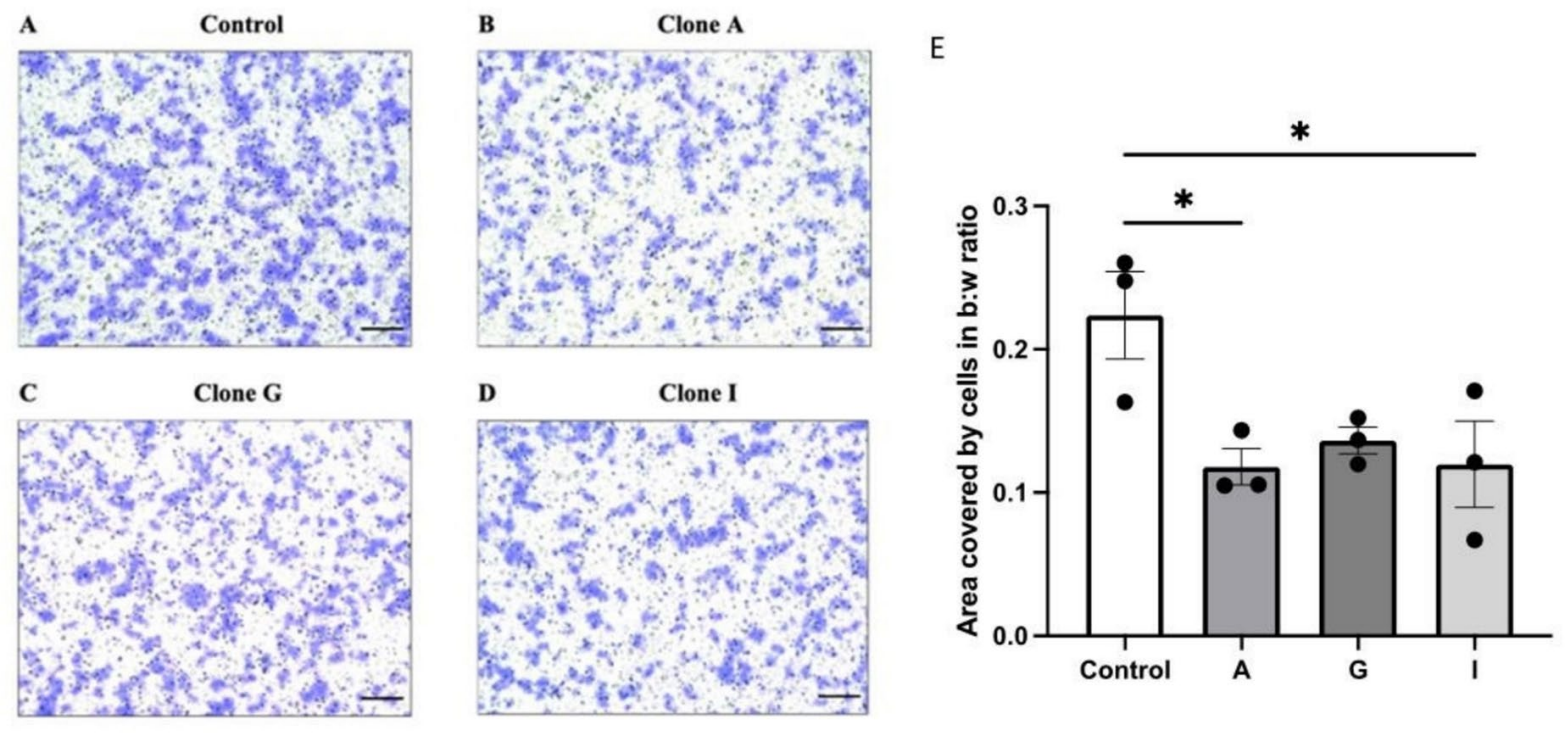
Stable αENaC overexpression reduced cell migration in a Boyden Chamber assay. Cells were seeded above a porous membrane in a Boyden chamber and left to migrate for 5 h. **A**–**D** Representative images of control, A, G, and I clone MDA-MB-231 breast cancer cells post-staining and fixing after 5-h incubation. Scale bar 200 μm. **E** Reduction in the number of cells migrated in the 5-h incubation in all three clones compared to the control cells, significant reduction for clones **A** and **I**. Eight images were taken per well and each cell type was run with two technical repeats. Images were turned to black (B) and white (W) pixels and the B:W ratio was determined using ImageJ, with the average of the eight images reported. One-way ANOVA with Dunnett’s post hoc test, resulted in a statistically significant difference, *p < 0.05, *N* = 3, *N* = 6. Data are shown as mean ± SEM

A reduction in cell migration with overexpression of αENaC was further confirmed using a Boyden chamber assay for migration. Figure 4 A shows that all three αENaC overexpressing clones had a reduction in the number of cells migrating through the chamber and adhering to the underside upon staining. There was a significant reduction in migration when comparing control to clones A and I (one-way ANOVA with post hoc tests, *p* < 0.05 (Fig. 4E, confirming that overexpression of αENaC resulted in reduced migration ability in independent clones of MDA-MB-231 cells.

### αENaC overexpression in MDA-MB-231 cells results in reduced proliferation

**Fig. 5:**
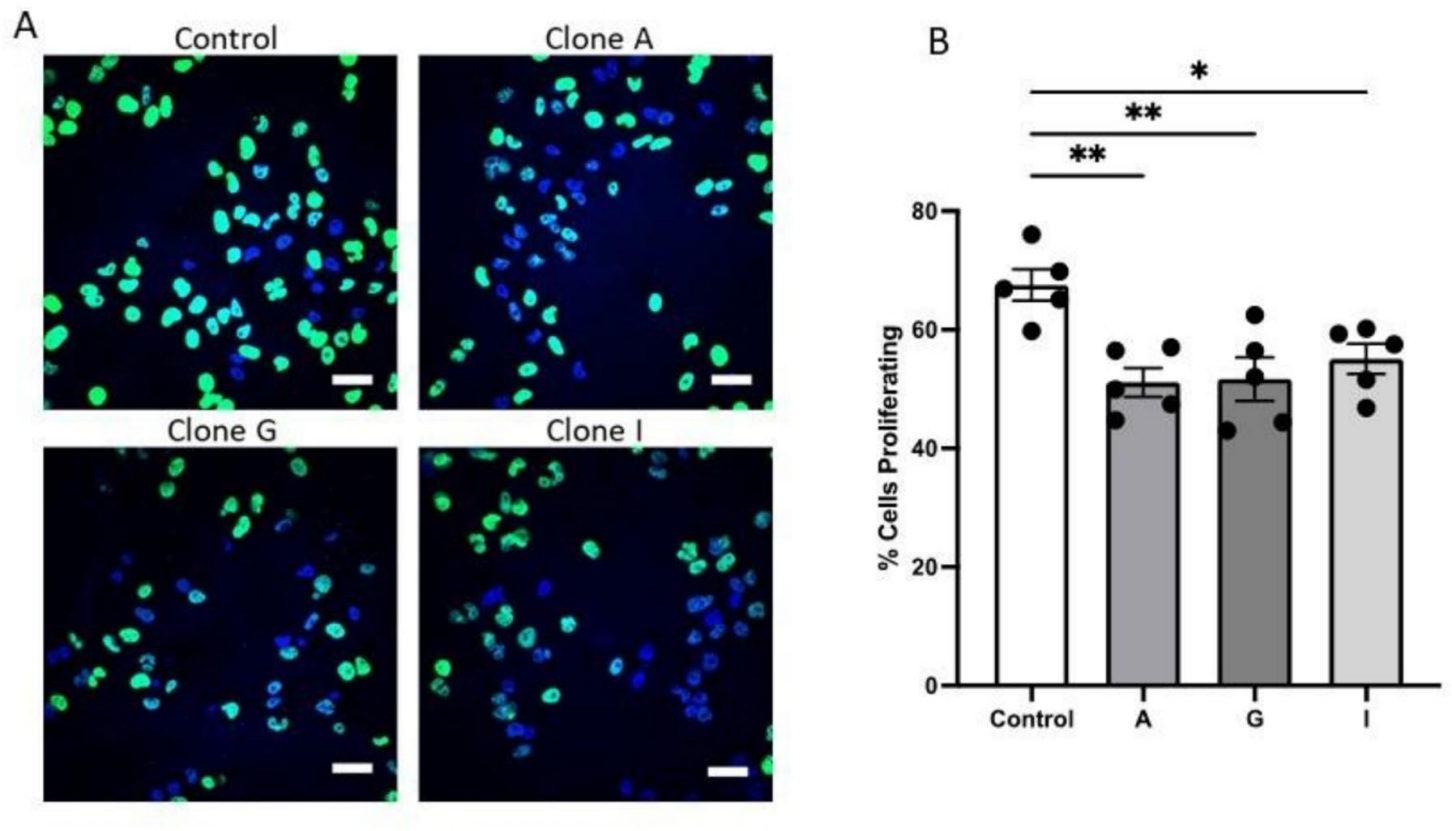
Stable αENaC overexpression reduces cell proliferation. MDA-MB-231 clones and control cells were incubated with EdU and prepared for imaging as per the manufacturer’s protocol for the EdU Click Kit. **A** Example images of control and three αENaC MDA-MB-231 overexpressing clones (A, G, I). Green is EdU (proliferating cells) and blue is the counterstain of DAPI (cell nuclei). Scale bar 50 μm. **B** Overexpression of αENaC in clones led to a significant reduction of breast cancer cells proliferating. One-way ANOVA with multiple comparisons to control, **p < 0.01, *p < 0.05. Data are shown as mean ± SEM, *N* = 3

Furthermore, as with migration, heightened proliferation is also seen during the metastatic cascade to enable the formation of a secondary tumour [27]. Our previous data showed a correlation between αENaC and proliferation markers as well as reduced proliferation following transient transfection of αENaC in breast cancer cell lines [18]; therefore, here we examine if this is also evident with stable αENaC overexpression. The stable overexpression of αENaC resulted in reduced proliferation, as visualized using an Edu assay, with the overexpressing clones all having fewer green cells and thus fewer cells that were undergoing proliferation (Fig. 5A). The overexpression of αENaC in all three of the clones resulted in an approximately 20% reduction in the number of cells proliferating (Fig. 5B), and this reduction was statistically significant when comparing each of the clones to the control cells (one-way ANOVA with post hoc test, *p < 0.5, **p < 0.01).

## Discussion

The epithelial sodium channel was originally recognized to have a key role in epithelia of the kidney, colon, and lung to reabsorb sodium and thus contribute to maintaining blood volume and pressure and the airway surface liquid layer in the lungs [28]. More recently, a potential role for epithelial sodium channels in regulating cancer cell functions such as proliferation and migration has been investigated in various types of cancer [12–18] and reviewed by Liu et al. [7]. However, there are currently only a limited number of papers that have examined the influence of ENaC in breast cancer cells [18, 19, 24, 29, 30]. In this study, we confirmed that αENaC protein levels were significantly lower in a TNBC cell line (MDA-MB-231 which has showed low endogenous ENaC expression) compared to a less aggressive cancer cell line (T-47D) (Fig. 2). Therefore in this study we aimed to provide insight into the role ENaC may have in cancer development and metastasis through bioinformatic analysis and through stably increasing the expression of the αENaC subunit in the advanced TNBC cell line MDA-MB-231 followed by assessing the ability of these breast cancer cells to proliferate and migrate. We hypothesised that upregulation of αENaC would decrease both cell migration and proliferation.

Our bioinformatic analysis, using publicly available data on KM plotter [21, 22], showed that all four ENaC subunits are expressed in breast tissue, matching studies from others reporting that ENaC subunit mRNA is expressed in mammary epithelial cells, both normal and cancerous [18, 29, 30]. Figure 1 shows that higher expression of αENaC and δENaC but not the β- and γ- subunits of ENaC correlated with better prognosis. This result aligned with our previous findings using a different dataset [18].

To investigate a link between αENaC expression with cell migration-associated genes, we used the SCAN-B dataset and our previous methods [18]. We found that higher expression of αENaC correlated with lower expression of 96 migration-associated genes in breast cancer patients. This supports our hypothesis that high levels of αENaC expression decrease levels of expression of genes known to be associated with metastasis: both proliferation-associated genes as previously reported [18] and migration-associated genes reported here in Fig. 1E. However, increased αENaC expression did not significantly alter mRNA or protein levels of the E-cadherin, N-cadherin, or vimentin EMT markers.

We then created three independent MDA-MB-231 cell lines stably overexpressing αENaC, and confirmed overexpression of αENaC at the mRNA and protein level (Fig. 2). Therefore, the results facilitate the forming of the hypothesis that the expression of ENaC, particularly αENaC, would be decreased in more advanced breast cancer, and overexpressing αENaC could impact malignant phenotypic behaviour.

In vitro assays were then used to address whether stable upregulation of αENaC influences breast cancer cell migration. Using both scratch assay (Fig. 3) and Boyden chamber assays (Fig. 4), we observed that all three αENaC overexpressing clones showed significantly reduced migration ability compared to the control cells, which aligned with our bioinformatic data of a negative correlation between αENaC expression and migration-associated genes. Using an EdU assay we showed that stable overexpression of αENaC significantly reduced cell proliferation, aligning with the results seen previously with transient αENaC overexpression [18].

There are several pathways through which overexpression of αENaC could exert an effect on breast cancer cells, resulting in reduced migration and proliferation. αENaC overexpression may interfere with intracellular positive ion concentrations interrupting Ca^2+^ signaling, as Ca^2+^ is required for normal and cancerous cell proliferation and migration [31, 32]. In particular, the additional sodium ions could interfere with the normal function of the sodium/calcium exchanger, causing it to change direction changing the cells’ calcium handling [33]. Cytoskeletal changes may occur due to the interaction of the C-terminal domain of αENaC with F-actin [34]. Increases in αENaC may interrupt the cell’s normal ability in reorganizing the cytoskeleton that is necessary for the breast cancer cells to achieve migration [35]. Slowing of the cells’ migration ability could also be due to changes in ion and water flow at the leading and lagging ends of the cell preventing the localized cell volume changes necessary for migration [36, 37]. Further research is required to determine exactly how the overexpression of αENaC resulted in changes in proliferation and migration and to identify which cellular pathways are changed.

## Conclusion

This work builds on our previous findings providing evidence that higher αENaC expression in cells that normally have reduced ENaC expression (more advanced BC cells) may be protective against cancerous proliferation and migration. Collectively, our results suggest avenues for future research to develop further our understanding of the role ENaC has in breast cancer cells. The work presented here provides further insight into the potential of targeting ENaC in developing novel breast cancer treatments.

## Supplementary Information

Below is the link to the electronic supplementary material.

Supplementary file1 (DOCX 670 KB)

## Data Availability

Gene expression analysis was performed using data from publicly available SCAN-B dataset and KM plotter.

## Abbreviations

DAPI: 4′ 6-Diamindine-2-phenylindole
EdU: 5-Ethynyl-2’-deoxyuridine
EMT: Epithelial–mesenchymal transition
ENaC: Epithelial sodium channel
OMNI: Otago Micro and Nanoscale Imaging Unit
TNBC: Triple-negative breast cancer
