# Supplementary material for: Stable overexpression of the epithelial sodium channel alpha subunit reduces migration and proliferation in breast cancer cells
Source: Breast Cancer Res Treat. 2025 Apr 12;211(3):595–604. doi: 10.1007/s10549-025-07667-w (PMC12031891; doi:10.1007/s10549-025-07667-w)
Supplement: Supplementary file 1 — Supplementary file1 (DOCX 670 KB) [file 10549_2025_7667_MOESM1_ESM.docx]

### Supplementary material for:

**Stable overexpression of the epithelial sodium channel alpha subunit reduces migration and proliferation in breast cancer cells.**

Sarah R.A. McQueen, Wey Qi Chin, Heather E. Cunliffe, Fiona J. McDonald

### Stable α-ENaC overexpression has no effect on the mRNA or protein levels of several EMP markers in the MDA-MB-231 breast cancer cells

**Methods**

**RNA extraction and RTqPCR:** Total RNA was extracted from cells using the PureLink® RNA Mini Kit (Cat No 12183018A, Life Technologies) following the manufacturer’s instructions. Total RNA was reverse transcribed using a commercial kit (PrimeScript, Medi’Ray, New Zealand). RNA (1 μg) and gene-specific primers (Sigma) were used for real-time PCR with SYBR Premix Ex Taq (Tli RNase H Plus) ROX plus (Medi’Ray), and a Bio-Rad CFX connect Real-Time system (BioRad). The sequences of primers used are as follows: *CDH1*, forward, 5’-CCGAGAGCTACACGTTC-3’, and reverse, 5’-TCTTCAAAATTCACTCTGCC-3’; *CDH2*, forward, 5’- ACATATGTGATGACCGTAAC-3’, and reverse, 5’- TTTTTCTCGATCAAGTCCAG-3’; *VIM*, forward, 5’- GGAAACTAATCTGGATTCACTC-3’, and reverse, 5’- CATCTCTAGTTTCAACCGTC-3’; TWIST1, forward, 5’-CTAGATGTCATTGTTTCCAGAG-3’, and reverse, 5’-CCCTGTTTCTTTGAATTTGG-3’; *SNAI1*, forward, 5’-CTCTAATCCAGAGTTTACCTTC-3’; and reverse 5’-GACAGAGTCCCAGATGAG-3’; GAPDH, forward, 5’-ACAGTTGCATGTAGACC-3’, and reverse, 5’-TTGAGCACAGGGTACTTTA-3’. mRNA expression was normalized to the internal control GAPDH. Data are represented as relative dCT expression.

**Western Blot**: Whole cell protein lysate was extracted using lysis buffer (TBS: 50 mM Tris, 150 mM NaCl (pH 7.4), plus 1% Triton X-100, 1 × Complete protease inhibitor cocktail (Roche)), and concentration was determined by DC Protein assay kit (Bio-Rad). Samples were separated by 10% SDS-PAGE and the protein was transferred to PVDF membranes (Sigma-Aldrich) for 2 hr at 45 mA (Hoefer Semiphor, Semi-dry transfer unit). Following blocking in 5% skim milk in wash buffer (TBS+ 0.1% Tween-20), the membrane was incubated with the appropriate primary antibody: αENaC (1:1000, cat no: SPC-403, StressMarq); E-cadherin (1:1000, cat 24E10, Cell Signalling Technology); N-cadherin (1:1000, cat no: D4R1H, Cell Signalling Technology); Vimentin (1:1000, cat no: D21H3, Cell Signalling Technology); or beta-actin (1:5000, cat no: Sigma-Aldrich) overnight at 4^o^C. After washing membranes were incubated with secondary antibodies (goat anti-rabbit-HRP (1:10000, cat no: A6154, Sigma-Aldrich) or goat anti-mouse-HRP (1:10000, cat no: A5441, Sigma-Aldrich)) for 1hr at room temperature. Protein bands were visualized using ECL (GE Healthcare) on a ChemiDoc^TM^ imager (Bio-Rad) or on CL-XPosure autoradiographic film (Thermo Scientific) and developed. To quantify the protein signal, densitometry was undertaken using ImageJ software and protein concentration was normalized to beta-actin.

### Stable α-ENaC overexpression has no effect on the mRNA level of EMP markers in the MDA-MB-231 breast cancer cells


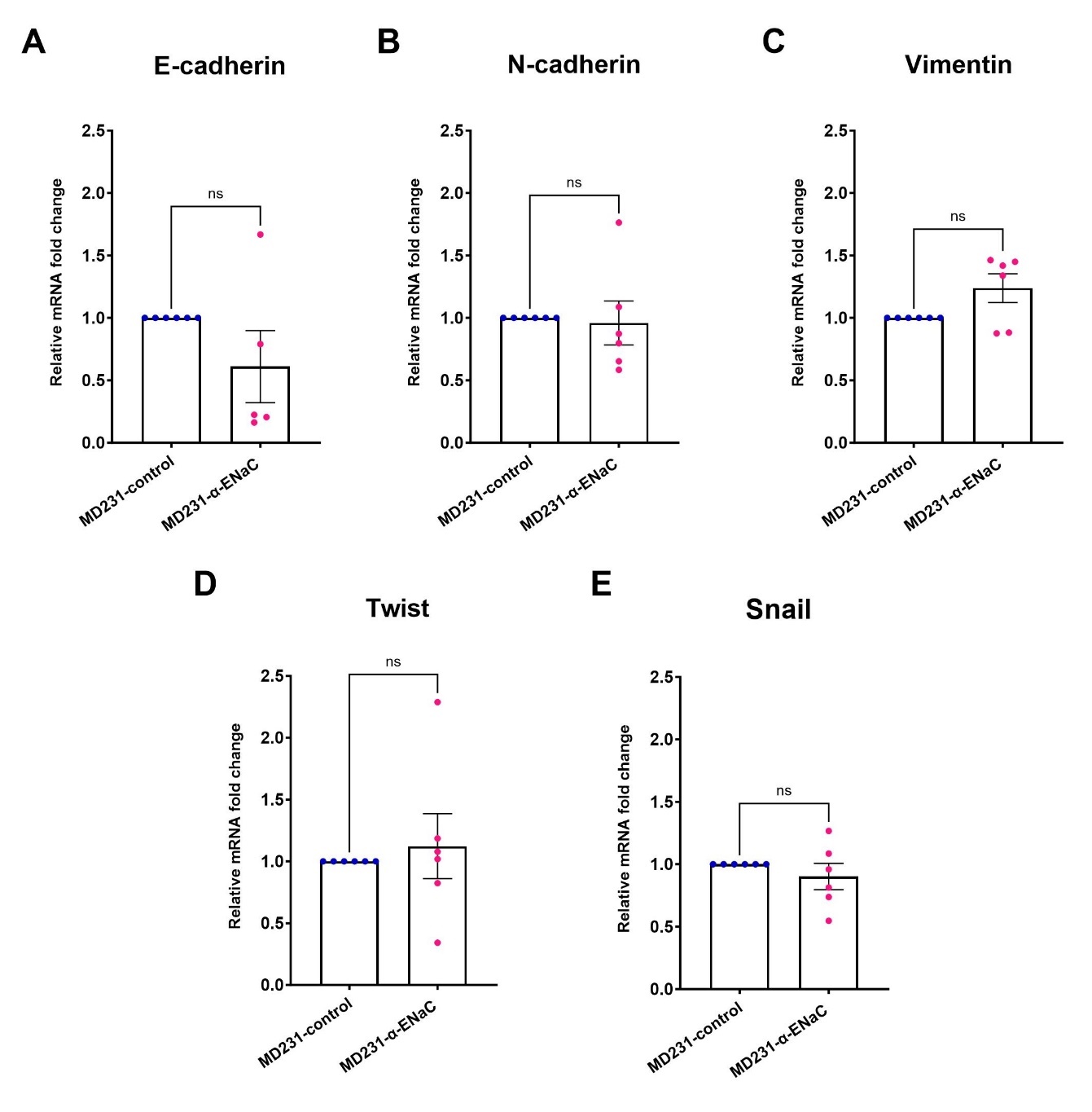


Figure 1. Stable α-ENaC overexpression does not affect the mRNA levels of EMP markers in the MDA-MB-231 cells.

Changes in the mRNA levels of **(A)** E-cadherin, **(B)** N-cadherin, **(C)** Vimentin, **(D)** Twist, and **(E)** Snail in the MD231-control and MD231-α-ENaC cells were analyzed with RT-qPCR. mRNA levels for each gene were normalized to GAPDH (reference gene) and shown as fold change relative to MD231-control cells (relative mRNA normalized to one). Data are expressed as mean ± SEM. Unpaired t-test with Welch’s correction, ns, not significant. The points on the graphs show six independent cell passages were used (N=6), and qPCR was performed in triplicate for the RNA samples from each cell passage, n=18 (triplicates for each N). **(A)** One outlier in the E-cadherin mRNA fold change data was removed using the Grubb’s test (alpha=0.05).

### Stable α-ENaC overexpression has no effect on the protein level of EMP markers in MDA-MB-231 breast cancer cells


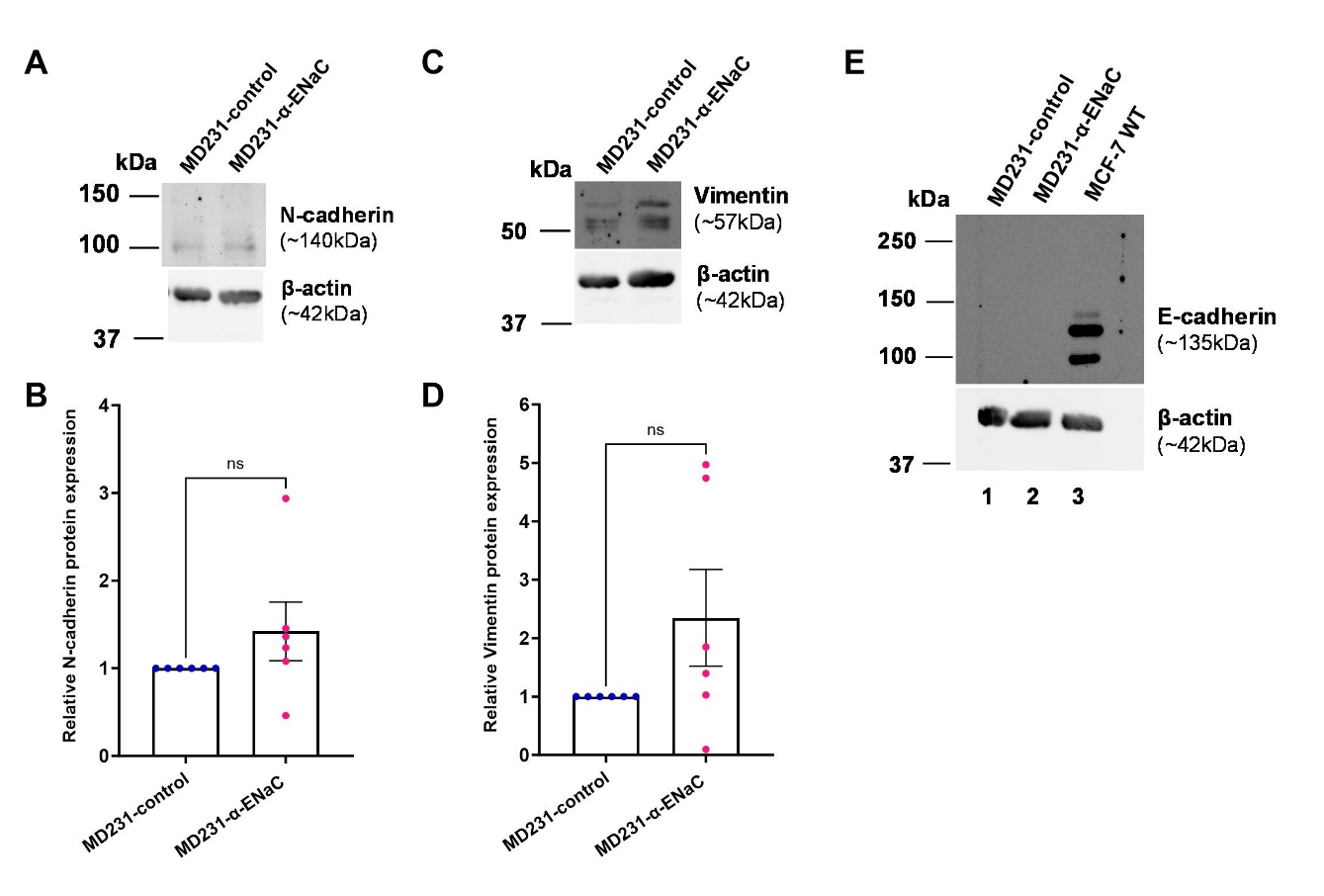


Figure 2. Stable α-ENaC overexpression does not affect the protein expression of EMP markers in the MDA-MB-231 cells.

**(A,C)** Representative western blot of **(A)** Vimentin, **(C)** N-cadherin, and β-actin (loading control) proteins in the MD231-control and MD231-α-ENaC cells. **(C)** N-cadherin protein was detected at ~100 kDa. **(B,D)** Density of protein bands were semi-quantified using ImageLab and then normalized to respective β-actin. **(B)** Vimentin and **(D)** N-cadherin protein expression is shown as mean ± SEM relative to the MD231-control cells (expression normalized to one). Unpaired t-test with Welch’s correction, ns, not significant. N=6, where the points on the graphs indicate biological repeats of six independent cell passages. **(E)** Representative western blots of E-cadherin and β-actin (loading control) proteins in the MD231-control, MD231-α-ENaC, and wild-type MCF-7 (positive control) cells. E-cadherin protein was not detected in the stable cell lines. N=1. WT, wild-type; kDa, kilodalton.
